# Do Power Meter Data Depend on the Device on Which They Are Collected? Comparison of Eleven Different Recordings

**DOI:** 10.3390/s25020295

**Published:** 2025-01-07

**Authors:** José-Antonio Salas-Montoro, Ignacio Valdivia-Fernández, Alejandro de Rozas, José-Manuel Reyes-Sánchez, Mikel Zabala, Juan-José Pérez-Díaz

**Affiliations:** Department of Physical Education and Sport, Faculty of Sport Sciences, University of Granada, 18011 Granada, Spain; salasmontoro@ugr.es (J.-A.S.-M.); alejandrodrg@correo.ugr.es (A.d.R.); jomaresan@correo.ugr.es (J.-M.R.-S.); mikelz@ugr.es (M.Z.); jjpd@ugr.es (J.-J.P.-D.)

**Keywords:** cycling, power, cadence, cycle computer, Garmin, all-out effort

## Abstract

This study evaluated the influence of cycle computers on the accuracy of power and cadence data. The research was divided into three phases: (1) a graded exercise test (GXT) at different constant loads to record power and cadence data; (2) a self-paced effort lasting 1 min to measure mean maximal power output (MMP); and (3) a short all-out effort. Eight cyclists completed the GXT, ten participated in the 1-min test, and thirty participated in the sprint effort. All participants pedaled on a controlled-resistance cycle ergometer, and the data were recorded using the ergometer itself and ten synchronized cycle computers of the same brand, configured to record at 1 Hz. The results showed minimal variations in power and cadence between devices during the GXT, suggesting adequate accuracy for constant efforts lasting a certain duration. However, in self-paced and high-intensity efforts (1-min and short all-out efforts), significant differences were observed between several devices, particularly in cadence and mean power, highlighting the relevance of device selection in these contexts. These findings suggest that, while variations in constant efforts may be negligible, in short-duration, high-intensity activities, the choice of device may be crucial for the accuracy and reliability of the data.

## 1. Introduction

Power is one of the most widely used metrics for evaluating performance in cycling, see, e.g., [1,2,3,4], both in training and in competition. Power measures the amount of work performed by the cyclist per unit of time, expressed in Watts, and has become a key indicator for planning specific training sessions, assessing effort, and quantifying fatigue [5,6]. The increasing availability of power meters on bikes has enabled athletes and coaches to monitor and adjust training loads based on power data [6,7,8].

Over the years, numerous devices have been validated for measuring power in cycling, such as laboratory ergometers and power meters installed on pedals, crank arms, or hubs. These devices provide accurate measurements under controlled conditions, and their reliability has been extensively studied [9,10,11,12,13,14]. However, although the accuracy of power meters has been the subject of exhaustive research, there is little information on how these data are recorded and stored. Laboratory ergometers, such as those used in scientific testing, usually have their own software that records power data—as well as other variables such as cadence—with high accuracy [9]. For this reason, it is not common to capture the signal—via ANT+ or Bluetooth—on a cycle computer. However, commercial power meters used in training and competition require another device to capture and store the output signal. The cycle computer is the most commonly used device in cycling for real-time display and recording data [2,3,4,11,15]. This data transmission process may be subject to variation due to multiple factors, such as signal quality, the communication technology used—ANT+ or Bluetooth—or the software of the cycle computer itself. These potential differences in how cycle computers record power data could significantly impact the reliability and accuracy of the information used to guide training decisions. However, to the best of our knowledge, no one has questioned this before. It is assumed that all cycle computers record data from different sensors, such as the power meter, in an identical way.

The present study aims to investigate whether power and cadence data vary depending on the cycle computer that records them. For this purpose, we intend to compare the data obtained with a laboratory ergometer with the record stored in ten different cycle computers. While previous research has focused on validating power meters and ergometers, this study addresses the relevance of the final device where the data are stored, a crucial yet underexplored aspect in daily cycling practice.

## 2. Materials and Methods

### 2.1. Experimental Design

The study involved three phases: first, power output and cadence data were collected from a cycle ergometer at different constant loads during a graded exercise test (GXT); second, the same data were recorded during a 1 min self-paced effort in which an attempt was made to obtain the mean maximal power output (MMP); and, finally, data were registered during a 5 s all-out effort.

### 2.2. Participants

The first experiment (GXT) involved 8 male cyclists (29.4 ± 10.8 years), the second experiment included 10 male cyclists (28.2 ± 9.9 years), and the last experiment was conducted with 30 cyclists (26.8 ± 8.3 years). All participants volunteered to take part in this study and provide written consent, in accordance with the ethical principles of the Declaration of Helsinki. The research was approved by the Local Ethical Committee of Research (1608/CEIH/2020).

### 2.3. Data Collection

In all three experiments, participants pedaled on a stationary ergometer (Atom X, Wattbike, Nottingham, UK). Power and cadence data were recorded with the cycle ergometer itself and, in turn, were transmitted via Bluetooth for recording on 10 cycle computers of the same commercial brand (Garmin, Lenexa, KA, USA): an Edge 1030, an Edge 1000, two Edge 830s, an Edge 820, an Edge 810, two Edge 530s, an Edge 520, and a Forerunner 735XT. All cycle computers were set to record at 1 Hz and synchronized at the start of each assessment.

Experiment 1 started with a 30 s pedaling phase at 50 W to allow the cyclist to adapt to his preferred cadence and to check that the signal was received on all devices. This was followed by 1 min steps at constant power, starting at 50 W and increasing by 50 W every minute, until the last step was completed at 400 W.

In experiment 2, after a 10-min free warm-up, the cyclists performed a 1-min self-paced effort with a standing start and were aiming to obtain their 1-min MMP. Finally, in experiment 3, after a 10-min free warm-up, cyclists completed an all-out effort lasting 3–5 s starting from a standing start.

### 2.4. Data Analysis

Open-source GoldenCheetah software (v.3.6) was used to retrieve the second-by-second power and cadence data from all devices and subsequently analyzed using a worksheet. Four variables were evaluated in the experiments: average power (P_AVG_), peak power (P_MAX_), average cadence (CAD_AVG_), and maximal cadence (CAD_MAX_). P_AVG_ and CAD_AVG_ were the average power and cadence over the entire time interval that was recorded, while P_MAX_ and CAD_MAX_ were the peak power and cadence values over that time interval. In experiment 1, these four variables were measured for each of the power steps in the GXT.

### 2.5. Statistical Analysis

The descriptive analysis included means and standard deviations (SD). To analyze the efforts at constant load during the GXT, a two-way—device and load—repeated-measures ANOVA was performed. The analysis included 11 levels for the device factor and 8 levels for the load factor. For the 1 min self-paced effort and for the all-out effort, a one-way repeated-measures ANOVA with 11 levels was performed. In all ANOVAs, sphericity was checked with Mauchly’s test, and the Greenhouse–Geisser correction was applied when this was considered violated (*p* < 0.05). When significant differences were found, post hoc comparisons were conducted using the Bonferroni correction to account for multiple comparisons. Statistical calculations were conducted using the open-source project JASP (version 0.19.1), with statistical significance accepted when *p* ≤ 0.05.

## 3. Results

### 3.1. Constant-Load Effort

Table 1 shows the power and cadence data for every device at each of the GXT steps. For P_AVG_ data at constant load, there were no significant differences for either the recording device (F(10, 70) = 0.896; *p* = 0.541; $\eta_{p}^{2}$ = 0.114) or the interaction between device and load (F(70, 490) = 0.803; *p* = 0.872; $\eta_{p}^{2}$ = 0.103). There were also no differences in the P_MAX_ data or the interaction between device and load (F(10, 70) = 0.916; *p* = 0.568; $\eta_{p}^{2}$ = 0.121). However, the devices did show differences between them (F(10, 70) = 3.997; *p* < 0.001; $\eta_{p}^{2}$ = 0.363). Post hoc comparisons revealed that the differences were between the Forerunner 735XT and the Wattbike (mean difference = 13.5 W; 95% CI [5.6–21.3]; *p* < 0.001; d = 0.459), the Edge 1000 (mean difference = 7.9 W; 95% CI [0.0–15.7]; *p* = 0.048; d = 0.269), and both Edge 530 devices (Edge 530(1): mean difference = 8.5 W; 95% CI [0.7–16.4]; *p* = 0.019; d = 0.291 and Edge 530(2): mean difference = 8.6 W; 95% CI [0.8–16.5]; *p* = 0.017; d = 0.224). There were also differences between the Edge 830(2) and the Wattbike (mean difference = 8.8 W; 95% CI [1.0–16.7]; *p* = 0.012; d = 0.302).

For cadence data at constant load, there were also differences between devices for both CAD_AVG_ (F(10, 70) = 72.645; *p* < 0.001; $\eta_{p}^{2}$ = 0.912) and CAD_MAX_ (F(10, 70) = 3.570; *p* < 0.001; $\eta_{p}^{2}$ = 0.338). Post hoc analysis showed the device differences reported in Table 2 and Table 3 (CAD_AVG_ and CAD_MAX_, respectively).

### 3.2. Self-Paced Effort

**Figure 1:**
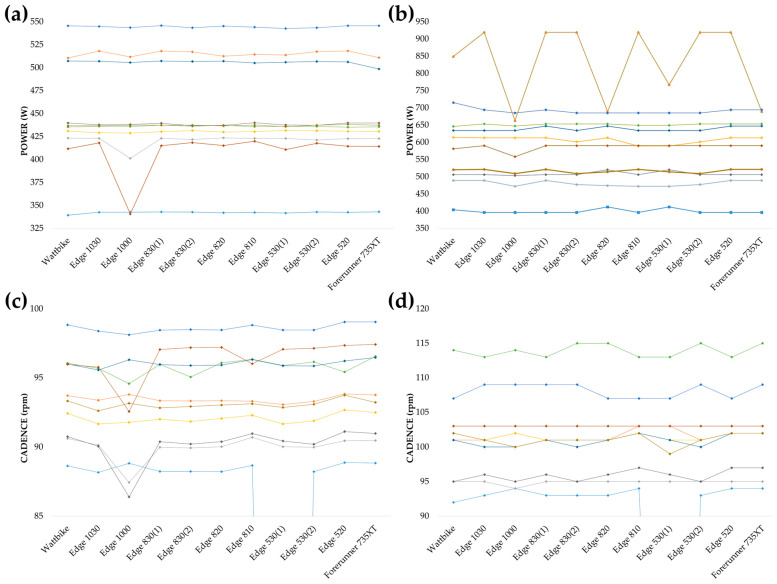
Individual data recording with each device for every one of the ten participants in the 1 min self-paced effort trying to reach their mean maximal power. (**a**) Values of average power; (**b**) values of peak power; (**c**) values of average cadence; and (**d**) values of maximal cadence.

There were no significant differences between devices for any of the variables in the 1 min self-paced effort: P_AVG_, F(10, 90) = 1.881, *p* = 0.058, $\eta_{p}^{2}$ = 0.173; P_MAX_, F(10, 90) = 1.098, *p* = 0.372, $\eta_{p}^{2}$ = 0.109; CAD_AVG_, F(10, 90) = 1.027, *p* = 0.428, $\eta_{p}^{2}$ = 0.102; and CAD_MAX_, F(10, 90) = 1.081, *p* = 0.385, $\eta_{p}^{2}$ = 0.107. Figure 1 shows the individual log data.

### 3.3. All-Out Effort

**Figure 2:**
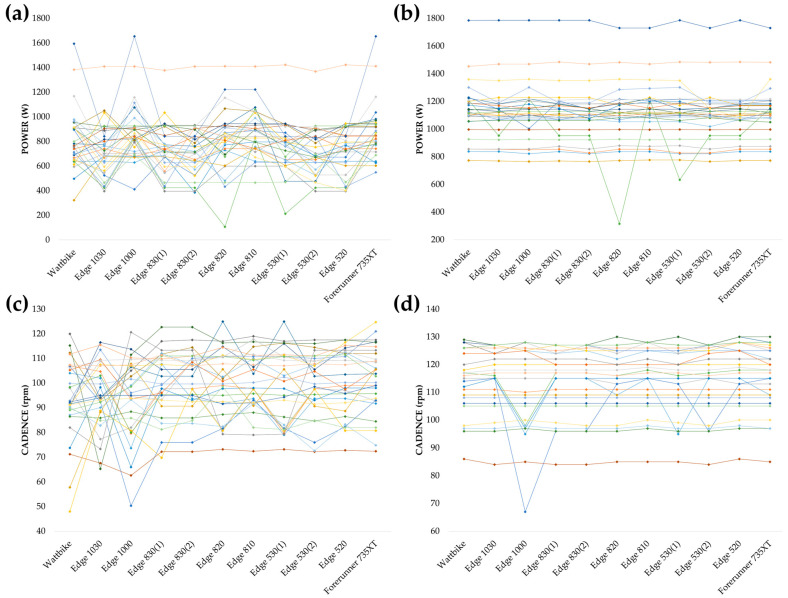
Individual data recording with each device for every one of the thirty participants in the short and all-out effort. (**a**) Values of average power; (**b**) values of peak power; (**c**) values of average cadence; and (**d**) values of maximal cadence.

In the short all-out efforts, no statistical differences were found in the P_MAX_ (F(1.594,46.216) = 1.304; *p* = 0.276; $\eta_{p}^{2}$ = 0.043) and CAD_MAX_ (F(1.613,46.787) = 2.448; *p* = 0.108; $\eta_{p}^{2}$ = 0.078) records. Nevertheless, there were significant differences in the P_AVG_ (F(3.821,110.814) = 6.422; *p* < 0.001; $\eta_{p}^{2}$ = 0.181) and CAD_AVG_ (F(4.349,126.118) = 3.276; *p* = 0.011; $\eta_{p}^{2}$ = 0.101) data. For CAD_AVG_, post hoc comparisons revealed that the differences were between the Forerunner 735XT and the Wattbike (mean difference = 7.9 rpm; 95% CI [0.4–15.3]; *p* = 0.025; d = 0.573), and between the Forerunner 735XT and the Edge 1000 (mean difference = 7.6 rpm; 95% CI [0.2–15.1]; *p* = 0.039; d = 0.553). For P_AVG_, post hoc analysis results are reported in Table 4. Figure 2 shows the individual log data.

## 4. Discussion

The purpose of this study was to compare the power and cadence recordings obtained with 11 different devices from the same signal from a cycle ergometer. To date, numerous studies have focused on the validation of cycle ergometers and power meters [9,10,11,12,13], i.e., the device that measures power and cadence and transmits the signal. However, we believe that it is assumed that all devices that receive the signal interpret it in the same way, as we are not aware of any previous studies that have examined this question.

The results of this study indicate that, although differences in power and cadence data recorded by various cycle computers are not always statistically significant, they do show practical variations in high-intensity, short-duration situations. This suggests that the choice of recording device can impact the precision and consistency of performance data, especially during short, intense efforts where variability between devices is amplified. The results of this study indicate that, as an effort is prolonged over time at constant intensity, the devices tend to yield the same mean power and cadence values. However, in self-regulated efforts, the shorter the duration and the higher the intensity, the more the differences tend to increase. This suggests that the choice of recording device may influence the accuracy and consistency of performance data, especially during short, intense efforts where inter-device variability is amplified. Similarly, it also calls into question previous validation studies of power meters and cycle ergometers, in which the differences between devices increased with higher power (e.g., [9,15]).

### 4.1. Constant-Load Effort

Although there were no statistically significant differences for P_AVG_ in the load x device interaction (*p* = 0.872) or for the device (*p* = 0.541), an examination of Table 1 shows that the values were not the same between devices, differing by 1 W at low loads and by 3 W at high loads. Although these minimal differences which are not statistically significant are not significant in practice [16], it is remarkable that they appear when the output signal received by the different devices is the same. Also in the case of the P_MAX_ achieved during each step of the GXT, no differences were found in the interaction between load and device (*p* = 0.568), but statistical differences between device measurements were reported (*p* < 0.001; $\eta_{p}^{2}$ = 0.363). Particularly noteworthy was the case of the Garmin Forerunner 735XT, which showed differences with up to four devices: a mean difference of 13.5 W compared to the measure of the Wattbike ergometer (*p* < 0.001; d = 0.459), a mean difference of 8.5 W and 8.6 W compared to both the Garmin Edge 530 devices (*p* = 0.019, d = 0.291 and *p* = 0.017, d = 0.224), and a mean difference of 7.9 W compared to the Garmin Edge 1000 (*p* = 0.048; d = 0.269). In addition, there was also a mean difference of 8.8W (*p* = 0.012; d = 0.302) between the ergometer reading and the value recorded by one of the Garmin Edge 830s. Although P_MAX_ is a point value and does not seem to be a relatively important difference in practical terms, these data corroborate that each device interprets the same signal differently. In this case, we were dealing with efforts at constant and relatively low load, but cycling is characterized by a great variability in efforts. For this reason, especially in the appearance of high values, these initial findings give rise to some doubt as to the effect that the type of device may have on the values we record.

For cadence, the ANOVA revealed statistically significant differences in average and maximal values between devices (*p* < 0.001, $\eta_{p}^{2}$ = 0.912 and *p* < 0.001,$\eta_{p}^{2}$ = 0.338 in both cases, respectively). However, post hoc comparisons (Table 2 and Table 3) showed that the mean differences were minimal (0.4–0.6 rpm), suggesting that, while the differences are statistically significant, they may not be practically meaningful in terms of cycling performance [16]. This statistical significance is likely to be due to the low variability of the data between devices, which allows very small differences to be detected as significant. In fact, looking at Table 1, the average values and standard deviation for each load among the different devices are practically the same in many cases. Subsequently, in Table 2, it appears that we could group the devices into two large groups that differ from each other by 0.5 rpm. The Edge 1000, the Edge 810, the Edge 520, and the Forerunner 735XT tend to measure an average of 0.5 rpm less than those devices in the other group formed by the Wattbike itself, the Edge 1030, both Edge 830s, the Edge 820, and both Edge 530s.

### 4.2. Self-Paced Effort

In contrast to what occurred with the GXT, neither of the variables showed statistically significant differences in the self-paced 1 min efforts attempting to achieve the MMP: P_AVG_, *p* = 0.058; P_MAX_, *p* = 0.372; CAD_AVG_, *p* = 0.428; and CAD_MAX_, *p* = 0.385. However, examining the individual data presented in Figure 1, it can be appreciated that, in some cases, the differences between devices are really important in practical terms. In particular, one of the most remarkable situations was that of a cyclist whose P_AVG_ with the Garmin Edge 1000 was between 75 W and 80 W lower than that recorded with the other devices. Continuing with the P_AVG_, there was also another cyclist whose value recorded with the Edge 1000 was more than 20 W lower than that with the other devices. In the case of the P_MAX_, the cyclist who reached a higher value (919 W measured with six devices) was very notable, as the difference between devices reached more than 200 W, with the Garmin Edge 1000, Edge 820, and Forerunner 735X providing the lowest values. In addition, the six devices that recorded 919 W provided a value 70 W higher than that indicated by the Wattbike itself. Finally, it can be appreciated that the cadence data also show some variability between devices, with one of the measurements of the first Edge 530 being particularly striking, in which, despite measuring power, the cadence data indicated 0 at all times during the minute of recording. Following the line of argument outlined above and as described by Atkinson [16], in this case, the absence of statistical significance does not imply that there are no practical differences in actual performance. This becomes even more relevant in competitive cyclists, where differences of a few Watts during short-duration efforts could significantly affect the outcome in competitive settings where every second counts [17]. Some studies reflect the impact of power measurement accuracy on decision making in competition [18,19]. The data obtained suggest that the choice of cycle computer should be considered when analyzing short, high-intensity efforts, where data variability is more likely and could influence final performance.

### 4.3. All-Out Effort

Finally, the short all-out efforts analysis showed significant differences in P_AVG_ (*p* < 0.001; $\eta_{p}^{2}$ = 0.181) and CAD_AVG_ (*p* = 0.011; $\eta_{p}^{2}$ = 0.101) between devices. For P_AVG_, post hoc analysis revealed that the differences were greater than 100 W between many of the devices (Table 4), which makes them very important differences in the practice of sports. The fact that there are such large differences in P_AVG_ and no statistically significant differences in P_MAX_ (*p* = 0.276) is due to data loss in some devices. Since these were short sprints, the failure of a device to collect 1 of the 3–5 samples greatly affected the average. However, the individual data (Figure 2) also show some cases with quite some variability for P_MAX_, such as the case where one of the cyclists recorded 315 W with the Edge 820, 633 W with one of the Edge 530s, and more than 1200 W with several devices. This finding reinforces the idea that the ability of devices to accurately record real-time data is particularly sensitive to differences during explosive efforts, characterized by rapid changes in power and cadence [15]. This information is particularly relevant in contexts where cyclists base their tactical decisions on prior training data or power profiles [4], as well as in certain cycling disciplines such as BMX racing or track speed events, in which short efforts are made at high intensities. In addition, this raises questions about other aspects, such as previous validation studies that indicated that the power meters differed in their data during sprint efforts [15,20]. With the data obtained in our study, we cannot deny that there are differences between the power meters, but perhaps they may be due to the device with which they were recorded.

### 4.4. Practical Applications, Limitations of the Study, and Future Research Directions

In validation studies on devices such as power meters, even if there is good agreement, it is usually indicated that it is not recommended to exchange data between devices [11,21]. This is common for professional cyclists with several bikes, as they have a power meter on each bike and should adjust their training values to each device. However, we add a new element that can interfere with the measurement: the cycle computer. Therefore, it is recommended not to interchange these devices either and to be consistent in the measurement, always using the same cycle computer with the same power meter. This is highly relevant for cyclists and coaches, as training planning and performance monitoring are carried out based on the power data recorded on the cycle computers. Consequently, erroneous readings due to the exchange of devices can lead to incorrect training loads or misinterpretations of performance.

In any case, this study has simply corroborated that there are differences between the measurements provided by different cycle computers of the same signal under controlled laboratory conditions. In future studies, it would be necessary to evaluate the reproducibility of the same device, to assess whether it is consistent in the data it provides. Likewise, we have only evaluated devices of a single commercial brand—Garmin. It would be interesting to compare devices from different brands commonly used in cycling such as Wahoo, Sigma, or Bryton. Their inclusion will allow a more comprehensive assessment of data accuracy across a broader range of devices, strengthening the results obtained and providing more generalizable conclusions. Likewise, it would be interesting to assess what happens in real field conditions, where variability due to different factors may influence the accuracy of the computers.

As a limitation of the study, especially in experiments 1 and 2, it should be noted that the sample size was small; therefore, in future proposed studies, it would be interesting to use a larger number of participants.

## 5. Conclusions

In conclusion, the results of this study suggest that, while differences in power data recording between devices may be minimal under steady-load conditions, in short, intense-effort situations, these differences could influence athletic performance and training planning. As noted in previous research, the reliability of power data is essential for cyclists and coaches seeking to maximize performance and adjust training loads precisely [22]. This study underscores the importance of standardization in data-capture systems and opens the door to future research on how cycle computers could be improved to provide more consistent and precise power data across a variety of sports conditions.

## Figures and Tables

**Table 1:** Average values and standard deviation of P_AVG_, P_MAX_, CAD_AVG_, and CAD_MAX_ data recorded with each of the devices at the different constant-load steps.

	Step (W)	Wattbike	Edge 1030	Edge 1000	Edge 830(1)	Edge 830(2)	Edge 820	Edge 810	Edge 530(1)	Edge 530(2)	Edge 520	Forerun. 735XT
**P_AVG_ (w)**	**50**	50.0 ± 1.2	49.7 ± 2.0	49.5 ± 2.5	50.3 ± 1.0	50.3 ± 0.9	50.2 ± 0.8	50.3 ± 0.9	50.1 ± 0.8	50.3 ± 0.8	49.6 ± 1.6	50.7 ± 1.4
	**100**	101.0 ± 2.2	101.1 ± 2.3	101.1 ± 2.1	101.1 ± 2.1	100.9 ± 2.1	101.1 ± 2.0	100.9 ± 2.5	101.1 ± 2.4	101.0 ± 2.3	100.9 ± 2.5	100.8 ± 2.6
	**150**	150.0 ± 1.1	151.2 ± 2.1	150.9 ± 2.0	150.9 ± 1.8	150.8 ± 1.9	150.9 ± 1.8	151.1 ± 1.6	150.8 ± 1.9	150.9 ± 2.0	150.7 ± 2.0	150.6 ± 1.7
	**200**	200.7 ± 3.0	201.0 ± 2.5	200.4 ± 2.3	200.3 ± 2.5	200.3 ± 2.3	200.4 ± 2.1	200.7 ± 2.5	200.3 ± 2.6	200.5 ± 2.5	198.2 ± 5.1	200.2 ± 2.3
	**250**	244.6 ± 4.9	245.4 ± 4.9	244.8 ± 4.4	245.6 ± 4.5	245.2 ± 4.9	246.0 ± 4.2	245.1 ± 4.6	245.4 ± 3.9	245.5 ± 4.8	245.5 ± 5.0	245.7 ± 3.6
	**300**	293.4 ± 5.4	294.0 ± 5.7	294.0 ± 5.5	293.5 ± 4.4	294.1 ± 5.8	292.1 ± 7.7	293.5 ± 5.1	294.4 ± 5.2	293.6 ± 5.8	292.0 ± 8.3	292.8 ± 4.9
	**350**	338.9 ± 6.3	338.9 ± 6.2	339.6 ± 5.5	338.7 ± 5.5	339.3 ± 6.1	340.7 ± 6.7	338.8 ± 6.3	339.6 ± 5.9	340.1 ± 6.8	337.2 ± 11.1	339.9 ± 5.6
	**400**	382.2 ± 11.4	384.8 ± 9.6	384.2 ± 8.9	384.9 ± 9.0	384.4 ± 9.5	385.0 ± 9.3	385.5 ± 9.5	385.0 ± 9.7	383.9 ± 10.0	385.4 ± 9.4	383.6 ± 9.9
**P_MAX_ (w)**	**50**	80.0 ± 12.1	78.6 ± 13.5	78.4 ± 12.9	78.3 ± 14.0	77.8 ± 14.0	79.0 ± 12.9	79.3 ± 8.2	78.6 ± 13.2	78.5 ± 13.5	75.8 ± 9.1	77.3 ± 13.5
	**100**	162.9 ± 29.9	146.8 ± 20.8	153.3 ± 28.2	148.0 ± 21.9	147.1 ± 19.7	155.5 ± 25.1	145.5 ± 23.7	152.4 ± 26.4	156.4 ± 26.4	148.4 ± 20.7	144.5 ± 27.3
	**150**	206.5 ± 29.3	203.0 ± 21.9	204.3 ± 21.3	203.0 ± 21.9	199.6 ± 18.6	212.8 ± 17.8	209.8 ± 18.6	208.1 ± 22.5	208.3 ± 19.3	203.0 ± 13.4	196.3 ± 17.6
	**200**	262.5 ± 25.3	257.6 ± 24.3	254.5 ± 24.9	257.6 ± 24.3	254.5 ± 24.9	249.5 ± 21.3	255.1 ± 23.7	256.1 ± 22.0	251.9 ± 23.5	257.6 ± 24.3	246.0 ± 20.9
	**250**	302.6 ± 14.7	300.3 ± 22.7	300.3 ± 22.2	301.5 ± 21.7	301.0 ± 21.1	296.9 ± 16.1	299.9 ± 17.9	300.5 ± 22.5	299.6 ± 15.2	303.3 ± 21.2	289.6 ± 14.8
	**300**	396.3 ± 33.7	388.9 ± 38.2	392.9 ± 39.2	388.9 ± 38.2	391.6 ± 39.0	394.9 ± 38.8	395.4 ± 34.6	399.3 ± 37.0	394.1 ± 39.2	391.4 ± 37.5	385.6 ± 40.2
	**350**	435.4 ± 35.4	432.9 ± 42.4	432.9 ± 41.3	433.4 ± 42.2	430.1 ± 43.9	427.1 ± 34.0	429.9 ± 30.9	429.9 ± 33.0	431.8 ± 41.0	433.1 ± 41.9	417.6 ± 32.9
	**400**	533.9 ± 49.6	519.1 ± 42.0	519.0 ± 40.6	520.4 ± 40.3	507.5 ± 30.7	509.8 ± 32.2	514.4 ± 39.8	515.6 ± 47.3	520.8 ± 48.6	512.4 ± 41.3	515.5 ± 52.4
**CAD_AVG_ (rpm)**	**50**	85.1 ± 7.8	85.1 ± 7.8	85.5 ± 7.9	85.1 ± 7.8	85.1 ± 7.9	85.1 ± 7.8	85.6 ± 7.8	85.1 ± 7.8	85.1 ± 7.8	85.6 ± 7.8	85.7 ± 7.8
	**100**	84.1 ± 6.7	84.1 ± 6.8	84.6 ± 6.7	84.1 ± 6.8	84.1 ± 6.8	84.1 ± 6.7	84.6 ± 6.7	84.1 ± 6.8	84.1 ± 6.8	84.6 ± 6.7	84.6 ± 6.7
	**150**	82.9 ± 7.9	82.9 ± 7.9	83.4 ± 7.9	82.9 ± 7.9	82.9 ± 7.9	82.9 ± 7.9	83.4 ± 8.0	82.9 ± 7.9	82.9 ± 7.9	83.4 ± 7.9	83.3 ± 7.9
	**200**	83.9 ± 8.1	83.9 ± 8.2	84.4 ± 8.2	83.9 ± 8.2	83.9 ± 8.1	83.9 ± 8.2	84.4 ± 8.2	83.9 ± 8.1	83.9 ± 8.2	84.4 ± 8.1	84.4 ± 8.2
	**250**	83.4 ± 8.2	83.5 ± 8.2	84.0 ± 8.3	83.5 ± 8.2	83.4 ± 8.2	83.5 ± 8.2	84.0 ± 8.2	83.5 ± 8.2	83.4 ± 8.2	84.0 ± 8.3	84.0 ± 8.3
	**300**	85.2 ± 8.6	85.2 ± 8.6	85.7 ± 8.7	85.2 ± 8.6	85.2 ± 8.6	85.3 ± 8.6	85.7 ± 8.6	85.2 ± 8.6	85.3 ± 8.6	85.7 ± 8.6	85.6 ± 8.7
	**350**	88.7 ± 7.6	88.9 ± 7.6	89.4 ± 7.6	88.9 ± 7.5	88.9 ± 7.5	88.9 ± 7.5	89.4 ± 7.6	89.0 ± 7.5	88.9 ± 7.5	89.4 ± 7.6	89.5 ± 7.6
	**400**	90.1 ± 13.3	90.2 ± 13.1	90.8 ± 13.1	90.3 ± 13.0	90.3 ± 13.2	90.4 ± 13.2	90.5 ± 13.0	90.4 ± 13.1	90.3 ± 13.2	91.0 ± 13.3	90.7 ± 13.5
**CAD_MAX_ (rpm)**	**50**	89.5 ± 7.8	89.5 ± 7.7	90.0 ± 7.9	89.6 ± 7.7	89.5 ± 7.8	89.5 ± 7.8	90 ± 8.1	89.6 ± 7.7	89.5 ± 7.8	90.1 ± 7.9	90.0 ± 8.1
	**100**	87.5 ± 6.6	87.3 ± 6.7	88.0 ± 6.7	87.3 ± 6.7	87.3 ± 6.7	87.4 ± 6.8	88.1 ± 6.8	87.3 ± 6.7	87.4 ± 6.8	88.0 ± 6.7	87.8 ± 6.8
	**150**	87.0 ± 7.8	87.0 ± 8.0	87.4 ± 7.7	87.0 ± 8.0	87.0 ± 8.0	87.0 ± 8.0	87.1 ± 8.4	87.1 ± 7.8	87.0 ± 8.0	87.6 ± 7.7	87.3 ± 8.0
	**200**	87.3 ± 7.5	87.3 ± 7.9	87.8 ± 7.8	87.3 ± 7.9	87.3 ± 7.9	87.3 ± 7.9	87.6 ± 7.7	87.4 ± 7.7	87.1 ± 7.8	87.6 ± 7.7	87.8 ± 7.8
	**250**	86.9 ± 9.2	87.6 ± 8.4	88.0 ± 9.7	87.0 ± 9.0	87.4 ± 8.1	87.0 ± 9.0	88.3 ± 8.6	86.9 ± 9.2	87.3 ± 9.2	87.8 ± 9.4	88.0 ± 9.3
	**300**	92.9 ± 7.8	92.6 ± 7.4	93.0 ± 7.4	93.6 ± 8.4	92.4 ± 7.1	93.3 ± 7.8	93 ± 7.4	92.8 ± 7.3	92.5 ± 7.2	92.8 ± 7.2	92.8 ± 7.2
	**350**	95.3 ± 8.6	95.3 ± 8.9	96.0 ± 8.9	96.3 ± 9.2	95.3 ± 8.9	95.4 ± 8.9	95.9 ± 9	95.3 ± 8.9	95.5 ± 8.9	96.0 ± 8.9	96.1 ± 9.3
	**400**	100.9 ± 11.4	100.6 ± 11.5	100.9 ± 11.3	102.1 ± 11.4	100.6 ± 11.5	100.5 ± 11.6	100.9 ± 11.6	100.9 ± 11.4	100.6 ± 11.6	101.0 ± 11.4	101.0 ± 11.5

P_AVG_, average power; P_MAX_, peak power; CAD_AVG_, average cadence; CAD_MAX_, maximal cadence.

**Table 2:** Results of post hoc analysis of the device factor for average cadence.

	Edge 1030	Edge 1000	Edge 830(1)	Edge 830(2)	Edge 820	Edge 810	Edge 530(1)	Edge 530(2)	Edge 520	Forerun. 735XT
**Wattbike**	*p* = 1.000	***p*** ** < 0.001** **d = −0.075** **MD = −0.5**	*p* = 1.000	*p* = 1.000	*p* = 1.000	***p*** ** < 0.001** **d = −0.070** **MD = −0.5**	*p* = 1.000	*p* = 1.000	***p*** ** < 0.001** **d = −0.079** **MD = −0.6**	***p*** ** < 0.001** **d = −0.074** **MD = −0.5**
**Edge** **1030**		***p*** ** < 0.001** **d = −0.070** **MD = −0.5**	*p* = 1.000	*p* = 1.000	*p* = 1.000	***p*** ** < 0.001** **d = −0.066** **MD = −0.5**	*p* = 1.000	*p* = 1.000	***p*** ** < 0.001** **d = −0.075** **MD = −0.5**	***p*** ** < 0.001** **d = −0.070** **MD = −0.5**
**Edge** **1000**			***p*** ** < 0.001** **d = 0.069** **MD = 0.5**	***p*** ** < 0.001** **d = 0.069** **MD = 0.5**	***p*** ** < 0.001** **d = 0.067** **MD = 0.5**	*p* = 1.000	***p*** ** < 0.001** **d = 0.065** **MD = 0.5**	***p*** ** < 0.001** **d = 0.070** **MD = 0.5**	*p* = 1.000	*p* = 1.000
**Edge 830(1)**				*p* = 1.000	*p* = 1.000	***p*** ** < 0.001** **d = −0.064** **MD = −0.5**	*p* = 1.000	*p* = 1.000	***p*** ** < 0.001** **d = −0.073** **MD = −0.5**	***p*** ** < 0.001** **d = −0.068** **MD = −0.5**
**Edge 830(2)**					*p* = 1.000	***p*** ** < 0.001** **d = −0.065** **MD = −0.5**	*p* = 1.000	*p* = 1.000	***p*** ** < 0.001** **d = −0.074** **MD = −0.5**	***p*** ** < 0.001** **d = −0.069** **MD = −0.5**
**Edge 820**						***p*** ** < 0.001** **d = −0.062** **MD = −0.5**	*p* = 1.000	*p* = 1.000	***p*** ** < 0.001** **d = −0.071** **MD = −0.5**	***p*** ** < 0.001** **d = −0.066** **MD = −0.5**
**Edge 810**							***p*** ** < 0.001** **d = 0.060** **MD = 0.4**	***p*** ** < 0.001** **d = 0.065** **MD = 0.5**	*p* = 1.000	*p* = 1.000
**Edge 530(1)**								*p* = 1.000	***p*** ** < 0.001** **d = −0.070** **MD = −0.5**	***p*** ** < 0.001** **d = −0.065** **MD = −0.5**
**Edge 530(2)**									***p*** ** < 0.001** **d = −0.074** **MD = −0.5**	***p*** ** < 0.001** **d = −0.069** **MD = −0.5**
**Edge** **520**										*p* = 1.000

d, Cohen’s D effect size; MD, mean difference (in rpm).

**Table 3:** Results of post hoc analysis of the device factor for maximal cadence.

	Edge 1030	Edge 1000	Edge 830(1)	Edge 830(2)	Edge 820	Edge 810	Edge 530(1)	Edge 530(2)	Edge 520	Forerun. 735XT
**Wattbike**	*p* = 1.000	*p* = 0.111	*p* = 1.000	*p* = 1.000	*p* = 1.000	***p*** ** = 0.047** **d = −0.066** **MD = −0.5**	*p* = 1.000	*p* = 1.000	***p*** ** = 0.028** **d = −0.066** **MD = −0.5**	***p*** ** = 0.025** **d = −0.062** **MD = −0.4**
**Edge** **1030**		*p* = 0.068	*p* = 1.000	*p* = 1.000	*p* = 1.000	***p*** ** = 0.002** **d = −0.066** **MD = −0.5**	*p* = 1.000	*p* = 1.000	***p*** ** = 0.010** **d = −0.066** **MD = −0.5**	*p* = 0.239
**Edge** **1000**			*p* = 1.000	*p* = 0.416	*p* = 1.000	*p* = 1.000	***p*** ** = 0.036** **d = 0.068** **MD = 0.5**	*p* = 0.277	*p* = 1.000	*p* = 1.000
**Edge 830(1)**				*p* = 1.000	*p* = 1.000	*p* = 1.000	*p* = 1.000	*p* = 1.000	*p* = 1.000	*p* = 1.000
**Edge 830(2)**					*p* = 1.000	***p*** ** = 0.042** **d = −0.075** **MD = −0.5**	*p* = 1.000	*p* = 1.000	***p*** ** = 0.017** **d = −0.075** **MD = −0.5**	*p* = 0.312
**Edge 820**						*p* = 0.503	*p* = 1.000	*p* = 1.000	*p* = 0.284	*p* = 0.071
**Edge 810**							***p*** ** = 0.007** **d = 0.066** **MD = 0.5**	*p* = 0.062	*p* = 1.000	*p* = 1.000
**Edge 530(1)**								*p* = 1.000	***p*** ** = 0.004** **d = −0.066** **MD = −0.5**	*p* = 0.059
**Edge 530(2)**									***p*** ** = 0.027** **d = −0.071** **MD = −0.5**	***p*** ** = 0.007** **d = −0.066** **MD = −0.5**
**Edge** **520**										*p* = 1.000

d, Cohen’s D effect size; MD, mean difference (in rpm).

**Table 4:** Results of the post hoc analysis to find the P_AVG_ differences between devices in all-out efforts.

	Edge 1030	Edge 1000	Edge 830(1)	Edge 830(2)	Edge 820	Edge 810	Edge 530(1)	Edge 530(2)	Edge 520	Forerun. 735XT
**Wattbike**	*p* = 0.917	*p* = 1.000	*p* = 1.000	*p* = 0.564	*p* = 1.000	*p* = 1.000	*p* = 1.000	*p* = 0.310	*p* = 1.000	*p* = 1.000
**Edge** **1030**		***p*** ** = 0.004** **d = −0.640** **MD = −140**	*p* = 1.000	*p* = 1.000	*p* = 1.000	***p*** ** = 0.007** **d = −0.613** **MD = −135**	*p* = 1.000	*p* = 1.000	*p* = 1.000	***p*** ** = 0.002** **d = −0.657** **MD = −144**
**Edge** **1000**			***p*** ** = 0.009** **d = 0.604** **MD = 133**	***p*** ** = 0.002** **d = 0.668** **MD = 146**	*p* = 1.000	*p* = 1.000	*p* = 0.053	***p*** ** = <0.001** **d = 0.700** **MD = 154**	***p*** ** = 0.022** **d = 0.567** **MD = 124**	*p* = 1.000
**Edge 830(1)**				*p* = 1.000	*p* = 1.000	***p*** ** = 0.017** **d = −0.578** **MD = −127**	*p* = 1.000	*p* = 1.000	*p* = 1.000	***p*** ** = 0.006** **d = −0.621** **MD = −136**
**Edge 830(2)**					*p* = 1.000	***p*** ** = 0.004** **d = −0.641** **MD = −141**	*p* = 1.000	*p* = 1.000	*p* = 1.000	***p*** ** = 0.001** **d = −0.684** **MD = −150**
**Edge 820**						*p* = 1.000	*p* = 1.000	*p* = 0.891	*p* = 1.000	*p* = 1.000
**Edge 810**							*p* = 0.093	***p*** ** = 0.002** **d = 0.674** **MD = 148**	***p*** ** = 0.040** **d = 0.541** **MD = 119**	*p* = 1.000
**Edge 530(1)**								*p* = 1.000	*p* = 1.000	***p*** ** = 0.036** **d = −0.545** **MD = −120**
**Edge 530(2)**									*p* = 1.000	***p*** ** = <0.001** **d = −0.717** **MD = −157**
**Edge** **520**										***p*** ** = 0.015** **d = −0.584** **MD = −128**

d, Cohen’s D effect size; MD, mean difference (in W).

## Data Availability

The data that support the findings of this study are available on request from the corresponding author.
